# Blood product transfusion practices in pediatric critically ill patients at a tertiary care hospital, Pakistan

**DOI:** 10.12669/pjms.39.4.7503

**Published:** 2023

**Authors:** Muhammad Rafique, Sharmeen Nasir, Amber Kamran, Ammarah Jamal

**Affiliations:** 1 Muhammad Rafique, FCPS Pediatrics. Department of Pediatrics, Dr. RKMP Civil, Hospital Karachi, Dow University of Health Sciences. Karachi, Pakistan; 2 Sharmeen Nasir, FCPS Pediatrics. Department of Pediatrics, Dr. RKMP Civil, Hospital Karachi, Dow University of Health Sciences. Karachi, Pakistan; 3 Amber Kamran, FCPS Pediatrics. Department of Pediatrics, Dr. RKMP Civil, Hospital Karachi, Dow University of Health Sciences. Karachi, Pakistan; 4 Ammarah Jamal, FCPS Pediatrics. Department of Pediatrics, Dr. RKMP Civil, Hospital Karachi, Dow University of Health Sciences. Karachi, Pakistan

**Keywords:** Blood transfusions, Plasma, Platelets, Packed red cell, Critical Care

## Abstract

**Objective:**

To determine the frequency of inappropriate blood product transfusions in pediatric critically ill patients.

**Methods:**

We collected data for this descriptive study from January to December 2020 at the Pediatric Intensive Care Unit (PICU) of Dr. RKMP Civil Hospital, Karachi. We included all patients one month to 12 years of age, admitted to pediatric intensive care unit and received any blood product transfusion. We reviewed their medical records and noted the demographic and clinical variables, type of blood product transfused with its indication and determined whether transfusion was appropriate or not, as per the standard guidelines and consensus recommendations.

**Results:**

Number of patients who were transfused was n=39, out of which females were 51.3%. Mean age of the patients was 49.0 months ±50.9 months. Total number of transfusions done were n=135, with most common blood product transfused being Fresh Frozen plasma (FFP) in 44.4%. A total of n=117 (86.66%) transfusion were appropriate as per the standard guidelines, while 18 (13.33%) were inappropriate (P-value <0.5). The most common indication of FFP transfusion was INR >2.0 in 51.6%, for Packed red cell transfusion was hemoglobin between 5 and 7 gm/dl in 35.5% and for Platelets was <20,000 with risk of bleeding in 36.6%.

**Conclusion:**

This study highlights the occurrence of inappropriate transfusions in critically ill pediatric patients. We need to reinforce the knowledge of our health care workers and display the guidelines in intensive care units.

## INTRODUCTION

Transfusion of blood products is a key therapeutic modality and can prove lifesaving if performed as per specified indications.^1^ The frequency of transfusions in critically ill patients admitted in Pediatric intensive care unit (PICU) varies from 23.9% in India to 35.8% in Pakistan.^2^,^3^ The decision to transfuse is crucial because there are various complications associated with it which include transfusion transmitted diseases, ABO incompatibility, alloimmunization, bacterial contamination, gut ischemia, transfusion related acute lung injury (TRALI) and other immunomodulation phenomena.^1^,^3^-^5^ Various guidelines have been devised to guide transfusion practices and to outline indications of transfusions so that complications are avoided.^2^,^6^,^7^ Despite these guidelines and known complications of transfusion, there have been reports of unnecessary and inappropriate blood product transfusions i.e., transfusing blood components in absence of recommended indications.^1^,^2^,^4^,^8^

To our knowledge and literature search, there has been only a single study done in Pakistan to evaluate appropriateness of blood product transfusions in critically ill children in Rawalpindi, which showed significantly high percentages of inappropriate blood component transfusions.^8^ This data is important to reflect our current transfusion practices and to highlight areas of improvement, teaching and training. Keeping the scarcity of data available in Pakistan and significance of the inappropriate transfusions we designed this study to calculate the frequency of appropriate and inappropriate blood product transfusions in pediatric critically ill patients at a tertiary care hospital of Karachi, Pakistan.

## METHODS

This is an observational study conducted at the Pediatric Intensive Care Unit (PICU) of Dr. RKMP Civil Hospital Karachi, after taking approval from Institutional Review Board (IRB-1945) of Dow University of Health Sciences. On the basis of a previous study which shows the frequency of appropriate blood product transfusions to be 75.15%,^2^ 113 blood component transfusions were be required for this study at 95% confidence interval and absolute precision 8%, using OpenEpi version 3.03. We collected the sample from January to December 2020 by convenience sampling. We reviewed the files of all patients admitted in PICU during the study period and patients who were one month to 12 years of age, of both genders who received any blood product transfusion were included in the study.

Incomplete records and patients who had disorders requiring regular blood transfusions were excluded from the study. Data was extracted by the principal and co investigators and collected on a predesigned proforma which included clinical variables like age, gender, diagnosis, use of mechanical ventilation and inotropes, patient outcome, type and number of blood product transfusion and whether transfusion was appropriate or not. The transfusion which were performed as per the indications mentioned in the standard evidence-based guidelines for transfusion of blood products were labelled as appropriate transfusions, while those which were performed without standard indications mentioned in the guidelines were labelled inappropriate.^2^-^4^,^6^,^7^ All data was stored in a password protected computer and patient’s identifier was removed to maintain confidentiality. Data was entered and analyzed in SPSS version 22.0. Descriptive statistics were used. Frequencies and percentages were calculated for categorical variables and mean & standard deviation for continuous variables.

## RESULTS

A total of 135 transfusions were included in the study. Number of patients who were transfused was 39, out of which males were n=19 (48.7%) and females were 20 (51.3%). Mean age (standard deviation) of the patients was 49.0 months (50.9 months). There were 21 (53.8%) patients on mechanical ventilators and 20 (51.28%) on inotropes. Most of the patients got managed and discharged (N= 25; 64.10%), while 12 patients expired (34.28%) and one each was referred and left against medical advice.

**Fig.1:**
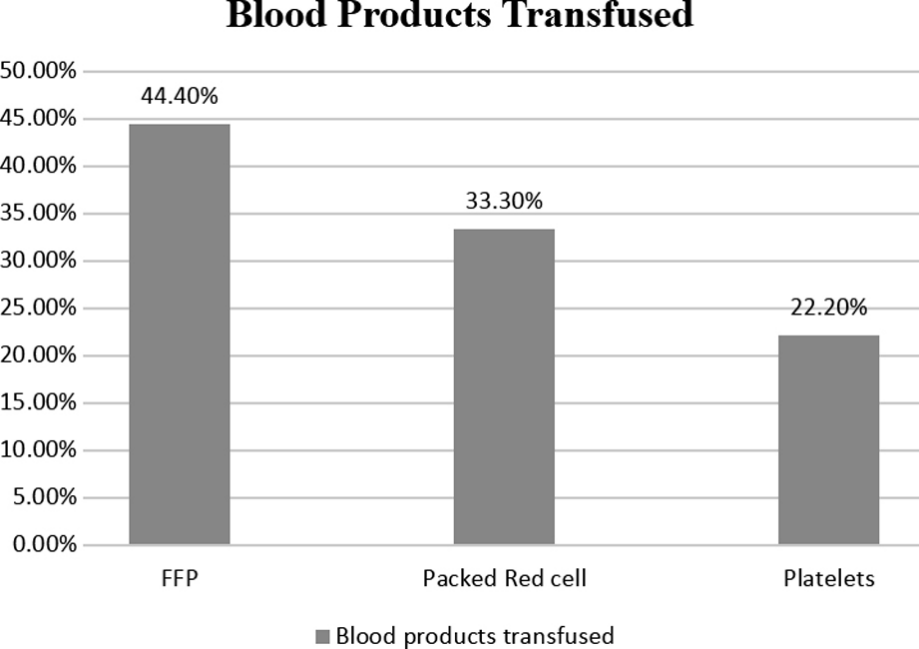
Frequencies of various blood products transfused.

Out of the total 135 transfusions, the most common blood product to be transfused was Fresh frozen plasma n=60 (44.4%), followed by Packed red cells 45 (33.33%) and Platelets 30 (22.22%) (Fig.1). The number of times Packed red cells were transfused shows a moderate but significant correlation with the times fresh frozen plasma is transfused (P <0.01).

Overall, there were 117 (86.66%) transfusions which were appropriate according to the standard guidelines of indications, while 18 (13.33%) were labelled inappropriate as they did not fit into any standard indication (P <0.5) (Table-I).

**Table-I:** Frequencies of appropriate and inappropriate blood product transfusions.

Blood product	Appropriate	Inappropriate	Total	P value
Packed Red Cells	36 (80%)	9 (20%)	45 (100%)	<0.5
Fresh Frozen Plasma	52 (86.5%)	8 (13.3%)	60 (100%)	
Platelets	29 (96.5%)	1 (3.3%)	30 (100%)	

Total	117 (86.6%)	18 (13.3%)	135 (100%)	

Among Fresh frozen plasma (FFP) transfusions, n=52 (86.66%) transfusions were appropriate and 8 (13.33%) were inappropriate. The most common indication of FFP transfusion was INR > 2.0 in 31 (51.6%) patients. Among Packed red cells transfusions, 36 (80%) transfusions were appropriate and 9 (20%) were inappropriate. The most common indication of Packed red cells transfusion was hemoglobin between five to seven gm/dl (N=16; 35.5%). Out of the 30 platelet transfusions, 29 (96.66%) were appropriate and one (3.34%) was inappropriate. The most common indication for platelets transfusion was platelet < 20,000 with risk of bleeding n=11 (36.6%). Details of indications are given in Table-II.

**Table-II:** Indications of transfusions of blood products.

Appropriate indication	Frequency (n)	Percentage (%)
***For Packed red cells:***		
Hemoglobin between 5 to 7gm/dl	16	35.55
Hemoglobin less than 5gm/dl	6	13.33
Hemoglobin between 7 to 10gm/dl with acute brain injury or congenital heart disease	10	22.22
Active massive bleeding	4	8.89
None of the above	9	20.00
***For Platelets:***		
Platelets < 50,000 and bleeding or invasive procedure	8	26.66
Platelets < 20,000 with risk of bleeding	11	36.6
Platelets < 10,000	1	3.33
Active massive bleeding	9	30.0
None of the above	1	3.33
***For Fresh frozen plasma:***		
INR > 2.0	31	51.67
DIC and bleeding	13	21.66
Active massive bleeding	8	13.33
None of the above	8	13.33

## DISCUSSION

Blood product transfusions have proved to be lifesaving in many of the life threatening situations and have given us time to get to the root cause and treat the disease. However, at the same time it is important to strictly follow the guidelines for indications, to avoid the risks of further harm and complications like infections, allergic and anaphylactic reaction etc. This study aimed to audit the blood product transfusion practices. In our study, we transfused a total of n=135 blood products, out of which 18 (13.33%) transfusions were inappropriate.

This frequency is less if we compare with the studies conducted in India where Sharif et al. reported 27.4% inappropriate blood product transfusions.^4^ The most common blood product transfused in our study was fresh frozen plasma (44.4%) and it was inappropriately transfused in 13.33%. Comparing this with other studies, the reported percentages of inappropriate fresh frozen plasma transfusion are 76% and 54.4% in India and Pakistan (Rawalpindi) respectively.^1^,^8^ Packed red cells were transfused in 33.3% of children out of which 20% were inappropriate. Again this frequency was low as compared to 26% reported by Jindal et al and 27.1% reported by Zafar etal.^1^,^8^ 22.2% of children were transfused platelets in our study out of which only 1 (3.33%) was transfused inappropriately as compared to other reports of 36.84%^4^ and 57%.^8^

The percentages of overall inappropriate transfusions as well as individual inappropriate blood product transfusion were lesser in our study as compared with other published literature, yet it exposes the patients to possible side effects as well as adds burden on the blood bank of a hospital where resources are already limited.

In our study, we found that the most common blood product inappropriately transfused was packed red cells (20%). The most plausible explanation for this is the confusion between newer restrictive vs. traditional liberal RBC transfusion strategy (<7.0 vs. <9.5g/dl). Studies indicate that pediatric intensivists have only partially adopted a restrictive transfusion strategy,^9^ despite randomized controlled trials (RCTs) indicating restrictive transfusion strategy to be similar in efficacy as compared to liberal strategy.^10^ Consensus recommendations for red cell transfusion practices have been published in 2018. They recommend restrictive transfusion strategies in settings of hemodynamically stable critical care patients with a threshold of 7gm/dl in general critically ill patients, patients of respiratory failure as well as sepsis and septic shock.^6^

Fresh frozen plasma (FFP) was transfused in 44.4% of patients. The commonest indication was INR >2.0. Eight transfusions (13.33%) of FFPs were inappropriate. Guidelines recommend not to use FFPs for volume replacement as the risks are greater than the benefits and not to correct INR 1.5-2 in a non-bleeding patient, not going for invasive surgery.^7^ Studies have shown that plasma transfusion does not correct mild to moderate coagulation abnormalities.^11^ Thus, this practice of using FFPs as volume expander or for correction of moderate coagulation abnormalities in absence of bleeding should be discouraged.

Platelets were the most appropriately transfused blood component in our study and only one (3.33%) of platelet transfusion was inappropriate. In contrast, other studies have shown a high rate of inappropriate platelet transfusions, as high as 57% in a study done in Pakistan.^8^ The most common indication for which platelets were transfused in our study was platelets < 20,000 with risk of bleeding (36.6%). This was similar to the findings of the study done by Sharif et al who also found the same most common indication for platelet transfusion.^4^ Other studies have shown that most of the platelet transfusions are done for prophylactic control of bleeding at counts more than 20,000, out of which many are significantly inappropriate.^12^,^13^,^14^

Advances in technology and screening have reduced the infectious complications of blood and blood product transfusions. However, it doesn’t exclude an individual from acquiring non-infectious complications which range from acute hemolytic transfusion reaction (AHTR), allergic reactions and anaphylaxis, transfusion related acute lung injury (TRALI) to alloimmunization and immunomodulation.^15^ A study conducted in China showed 1.35% of transfusion reactions in children over a period of five years, out of which main types of reaction were allergic and febrile non-hemolytic transfusion reactions.^16^ Moreover, there are studies which show that RBC transfusion in critically ill children is associated with non-favorable outcomes.^17^ Periodic clinical audits are an efficient tool to self-regulate the transfusion practices and avoid unnecessary use of blood products, hazard to patients and waste of resources.

### Limitations:

This study has some limitations. It is a retrospective study thus we don’t have the reasons of inappropriate blood product transfusion. It is a single-center study from a tertiary care, public sector hospital. The results may have been different in a private setup or secondary care hospitals.

## CONCLUSION

Our study demonstrates the presence of inappropriate blood product transfusion in the pediatric critical care unit of a tertiary care teaching hospital. This highlights the knowledge gap regarding indications of transfusion, which needs to be bridged as soon as possible to avoid unintentional harm to the patients.

### Recommendations:

We recommend and plan to re-inforce the knowledge of physicians and nursing staff of our institute regarding indications of various blood product transfusions and then perform this study again after some period of time to see the impact. Moreover, we recommend further prospective studies to assess the reasons of transfusions in settings where they were inappropriate and complications associated with them.

### Authors Contributions:

**MR:** Conceived the idea, reviewed the article critically and gave final approval.

**SN:** Interpreted the data, drafted the article and gave final approval. She is also responsible for the integrity and accuracy of the study.

**AK and AJ:** Contributed to acquisition of data, reviewed the article and gave final approval.

All authors agree to be accountable for the article.
